# Rabies glycoprotein engineering for improved stability and expression

**DOI:** 10.1016/j.vaccine.2025.127541

**Published:** 2025-07-26

**Authors:** Solomon English, Sofiya Fedosyuk, Francisco Orliacq, Vincent Tem, William Taylor, Nawsad Alam, Zhi Q. Xiang, Luke Thorley, César López-Camacho, Hildegund C. Ertl, Alexander D. Douglas

**Affiliations:** a Jenner Institute, University of Oxford, Old Road Campus Research Building, Roosevelt Drive, Oxford OX3 7DQ, United Kingdom; b Department of Biochemistry, University of Oxford, South Parks Road, Oxford OX1 3QU, United Kingdom; c The Wistar Institute, Philadelphia, PA 19104, USA; d Pharma V, Casablanca, Morocco

**Keywords:** Rabies virus glycoprotein, Structure-guided design, Subunit vaccine, Adenovirus, mRNA

## Abstract

Current rabies vaccines require multiple doses and are relatively expensive, limiting their accessibility. Novel low-cost vaccines capable of inducing a protective antibody response against the rabies virus glycoprotein (RVG) are therefore desirable. Structure-guided engineering of the antigen may enhance its qualitative or quantitative immunogenicity, as may transgene cassette optimisation in the case of vectored vaccines. We investigated the potential of these approaches for the design of improved rabies vaccines.

We evaluated twelve candidate cassette designs. While codon optimisation enhanced expression *in vitro*, it did not translate into improved immunogenicity. Co-expression or RVG with rabies matrix protein (RVM) did not detectably affect expression or immunogenicity. Inserting a C-terminal trimerisation domain was detrimental to expression *in vitro* and did not improve immunogenicity compared to the wild-type comparator. We screened 72 mutant constructs for *in vitro* expression and pre-fusion stabilisation. Several mutants enhanced expression and/or pre-fusion stability at low pH. Combination of the previously reported H270P mutation with the H419L substitution achieved enhanced stability. An L271Q + H419L double mutant achieved the greatest positive effect upon expression. Neither of double mutants improved immunogenicity compared to wild-type RVG when tested using an mRNA vaccine platform.

These mutant constructs may be of value for protein subunit vaccines, but full length wild-type RVG may be sufficiently conformationally stable and well-expressed for optimal immunogenicity of adenovirus and mRNA vaccines in mice.

## 1 Introduction

Rabies virus is a lyssavirus with a single-stranded RNA genome encoding only five proteins. As is characteristic of this genus, it is neurotropic and infects a wide range of mammalian hosts, though dogs are responsible for >99 % of human infections [1].

The virus infects cells of the central nervous system (CNS) after gaining entry to the body *via* skin puncture or contact with mucosal surfaces. Symptom onset precedes CNS infection from which point disease progression is almost universally fatal [2]. Rabies kills approximately 55,000 people each year, predominantly in Africa and Asia [3]. This situation persists despite rabies vaccines that have existed since the 19th Century and that induce well characterised protective immune responses [4].

Symptomatic disease is prevented by pre-exposure (PrEP) or post-exposure prophylaxis (PEP). PrEP comprises administering 2 or 3 doses of an approved vaccine over three weeks. For previously unvaccinated individuals, PEP consists of 3 to 5 doses of vaccine combined with a rabies antibody preparation; treatment must be initiated shortly after exposure [3]. Though these interventions save lives, they require repeated visits to a health clinic, and vaccination is often inaccessible for patients in low- and middle-income countries where the disease burden is greatest [5].

There remains significant incentive to improve rabies vaccines. Ideally, such vaccines would be inexpensive and induce a protective immune response after a single dose. Achieving this requires novel vaccines with improved immunogenicity. The rabies virus glycoprotein (RVG) is known to be the target of protective virus-neutralising anti-bodies (VNAs) and is thus the obvious immunogen for subunit vaccines [6]. Although it is important to note that some animals have been shown to be protected by previous vaccination (perhaps by an anamnestic recall response despite the absence of VNA at the time of challenge) [7], the acceptance of VNA titres above a threshold of 0.5 IU/mL as a surrogate for clinical protection makes VNA a reasonable assay for pre-clinical comparison of immunogenicity of candidate vaccines [3].

Numerous RVG-based novel rabies vaccines are in development, based upon a wide range of subunit vaccine platforms, and are potentially suitable for both human and veterinary applications. We have recently reported encouraging immunogenicity results of a single-dose adenovirus-vectored vaccine (‘ChAdOx2 RabG’) in a Phase I clinical trial [8], and clinical data have also been reported for a second adenovirus-vectored candidate [9]. Clinical data have been published for three mRNA-based rabies vaccines. Although immunogenicity improvement was apparent across progression from protamine-formulated to lipid-nanoparticle-formulated (LNP) versions of a nucleoside-unmodified candidate vaccine, and most recently a self-amplifying RNA candidate vaccine, none of them appeared to achieve durable seroconversion with a single dose [10–12]. Various recombinant protein / adjuvant-based vaccines have been described, including one which reached Phase III clinical trials [13].

RVG mediates infection of host cells [14] and recognises cell surface receptors to facilitate virus binding and membrane fusion [14,15]. Most neutralising antibodies target one of the three main antigenic sites (I, II, III) on RVG. RVG’s ability to adopt both pre- and post-fusion conformations may have implications for vaccine development should one conformation of the protein better present neutralising epitopes to the immune system. Interestingly, certain neutralising monoclonal antibodies such as RVC20, which targets antigenic Site I, exhibit specificity for the pre-fusion conformation [16]. However, it is unclear whether the pre- or post-fusion conformation might be better than the other at inducing VNAs.

Here, we describe two broad engineering strategies that sought to enhance immunogenicity of RVG-based vaccines. By increasing the quantity and/or quality of the antibody response raised by these vaccines, it is hoped that the number of doses required to surpass the accepted threshold for protection can be reduced, and that the protective period can be prolonged.

The first engineering strategy aimed to improve expression of the fully folded, trimeric RVG without modification of the ectodomain’s primary sequence (henceforth ‘wild-type RVG constructs’). This included comparisons between the wild-type (WT) antigen from the ERA strain and a codon-optimised sequence [17]; investigating different mammalian expression promoters; assessing whether incorporating C-terminal trimerisation domains could enhance RVG expression; and exploration of bicistronic/dual-antigen constructs where RVG was expressed in tandem with the rabies matrix protein (RVM). Regarding the latter, RVM is thought to associate with RVG [18,19], possibly *via* RVG’s C-terminal tail, assisting with its trafficking to the surface membrane and driving budding of particles resembling virions [20], and potentially contributing to RVG’s proper folding and expression. There is also evidence from a number of published studies that immunogenicity of nucleic acid-based vaccines (*i.e*. DNA, mRNA or viral vector) can be enhanced by addition to the antigen of a moiety promoting *in vivo* assembly of virus-like particles [21–23]. In the case of RVG, we therefore hypothesized that RVM could serve as such a moiety.

Following the limited benefits we observed when screening the WT RVG constructs, the second strategy we investigated sought to improve RVG’s immunogenicity through structure-guided mutagenesis (henceforth ‘ectodomain mutants’). This approach was inspired by recent successes in the development of pre-fusion stabilised vaccines against the fusogens of other enveloped viruses including respiratory syncytial virus (RSV) [24,25], Middle Eastern respiratory syndrome (MERS) [26] and SARS-CoV-2 [27].

Structure-guided design can improve levels of expression (a quantitative effect), stabilise a desired conformation (a qualitative effect), or both. Both such effects may be of relevance to recombinant protein and vectored vaccines (viral vector, RNA or DNA). In the case of vectored vaccines, the effect of expression changes or conformational stabilisation upon immunogenicity is not readily predictable and may vary between antigens and delivery methods.

Expression of a conformationally accurate antigen is important in inducing a protective immune response against RVG. Antigenic sites II and III (targeted by known nAbs) are largely comprised of conformational epitopes [6,28]. What is less clearly understood is whether the pre-fusion protein may be a better target for protective antibodies than the post-fusion conformation.

To investigate this, we describe the evaluation of a panel of 72 RVG mutants informed by a computational model of the pre-fusion RVG structure for the purpose of stabilising it.

## 2 Materials and methods

### 2.1 Molecular biology

The ‘ERA RVG’ construct under control of the LPTOS promoter is identical to that used in the previously described ‘ChAdOx2 RabG’ construct [8,29]. For all other ‘wild-type RVG’ constructs except for ‘PVoG RVG’, the WT RVG sequence is the same as in ChAdOx2 RabG. PVoG RVG is a codon-optimised chimera of the Pasteur Virus (PV) strain RVG ectodomain with the SAD-B19 strain intravirion sequence, previously designed to enhance RVG expression [17].

The WT ectodomain constructs were generated using standard molecular biology cloning techniques. RVG-encoding cassettes were inserted into pENTR4 plasmid backbones equivalent to the shuttle vector described previously for production of ChAdOx-vectored vaccines [29,30]. Details of restriction enzymes and cloning methods can be found in the supplementary material.

For the ectodomain mutant screens, the ‘WT’ RVG used as the baseline / comparator was PVoG. All sequences were synthesised by Twist Bioscience, pre-cloned into a pTT3 mammalian expression vector backbone described previously [31].

All RVG mutants are summarised in Supplementary Table S1, whilst combinatorial mutants can be found in Supplementary Table S2. The H270P mutant of RVG, as well as selected others, are protected under international patent filing WO2021181121A1.

### 2.2 Transient transfection of mammalian cells

Expi293F Cells (ThermoFisher: A14527) were cultured in Expi293 Expression Medium (ThermoFisher: A1435102) in a mammalian cell shaking incubator (SciQuip: SQ-4236) at 37°C, 8 % CO_2_, 80 % humidity, 130 rpm, 25 mm orbital diameter. Transfections were performed using cell cultures at a density of 3 × 10^6^ cells / mL and the ExpiFectamine transfection kit (ThermoFisher: A14524) as per the manufacturer’s instructions. The final plasmid DNA concentration used was 1 μg/mL in all cases. Except where otherwise indicated, transfected cultures were incubated for 4 days prior to harvest.

Unless otherwise stated, small-scale transient transfections of RVG and its mutants were performed in 96-well, square deep well plates (VWR: 732–3806), and at a volume of 600 μL using Expi293F cells by the same general method as above. Plates were sealed with Breathe-Easy sealing membranes (Merck: Z380059) and incubated at 37 °C, 8 % CO_2_ shaking at 900 rpm, 3 mm orbital diameter.

### 2.3 Protein purification

Antibodies were produced by transient transfection of Expi293F cells, as above, and purified using an AKTA Pure (Cytiva) and Pierce Protein G resin (ThermoFisher: 89927), as per the manufacturer’s instructions. Plasmid maps for the heavy- and light-chains of these antibodies can be found in the supplementary material.

Purified RVG protein used to coat ELISA plates and/or as a comparator (as stated in corresponding figure legends) was produced by transient transfection of Expi293F cells using plasmid DNA encoding the full-length WT RVG with a tetra-peptide C-tag (EPEA), and purified using CaptureSelect C-tag resin (ThermoFisher) as previously described [8].

### 2.4 Flow cytometry-based screening of wild-type RVG constructs

Expi293F cells, transfected with RVG construct-encoding plasmid DNA by the method described above, were stained for RVG in 96-well plates using 200 μL samples of transfected cells. Cells were pelleted at 1000 × G for 3 min and stained using three different methods. Total RVG surface expression was determined by staining cell pellets with polyclonal mouse sera from animals immunised intramuscularly with ChA-dOx2 RabG (5 × 10^5^ infectious units) [8] and boosted after 8 weeks with an inactivated rabies vaccine (Rabipur, 1/20 human dose), diluted 1:1000. Expression of RVG displaying neutralising antibody epitopes was measured by staining with two anti-RVG neutralising monoclonal antibodies: 17C7 (Site III) [32] and E559 (Site II) [33] at 1.2 μg/mL.

Stained cell pellets were washed in PBS, centrifuged as above and pellets were resuspended in a detection mix of 1 μg/mL anti-mouse IgG secondary antibody conjugated to allophycocyanin (ThermoFisher: A10539), diluted in PBS. Cell samples were incubated for 15 min, washed a final time, and analysed by flow cytometry on a BD LSR II flow cytometer (BD Biosciences).

Because a primary aim of the wild-type RVG strategy was to identify constructs with a potential to out-perform the transgene used in our clinical-stage adenovirus-vectored vaccine candidate (‘ChAdOx2 RabG’), the ‘baseline’ construct to which wild-type RVG candidates were compared was that used in ChAdOx2 RabG, comprising the ‘LPTOS’ intron-containing cytomegalovirus immediate-early promoter and ERA strain RVG sequence (without codon optimisation) (ClinicalTrials.gov ID: NCT04270838) [8].

### 2.5 Sandwich enzyme-linked immunosorbent assay (ELISA)

Concentrations of RVG variants in transfected cell lysates were quantified by a sandwich ELISA. ELISA plates (NUNC Maxisorp, ThermoFisher: 442404) were coated with one of two anti-RVG antibodies overnight at 2 μg/mL. The site-I-binding mAb SO57 recognises a linear epitope [34]. The site-III-binding mAb 17C7 recognises a conformational epitope [32].

Transfected cell lysates for each construct were added to wells coated with each of the aforementioned antibodies, their expression quantified by staining with polyclonal RVG-reactive mouse serum and detected using a goat anti-mouse IgG Fc-specific secondary antibody conjugated to alkaline phosphatase (Merck: A3562). Full method details are described in the supplementary information.

### 2.6 Detection of RVG-binding antibodies by ELISA

Total anti-RVG IgG titres from serum samples of immunised mouse were determined using a standardised ELISA format [35] and method described previously [8], with the modification that detection was performed with the alkaline phosphatase-conjugated goat anti-mouse IgG secondary described above. Blocking and washing steps were conducted as summarised in the supplementary methods. Anti-RVG titres were interpolated using a standard curve generated with a serum pool from mice immunised with inactivated rabies virus (IRV). Curve fitting and interpolation was performed using the MARS analysis suite (BMG Labtech).

### 2.7 Conjugation of amine-reactive fluorophores to antibodies

Direct labelling of antibodies with fluorophore was performed using AlexaFluor 647 NHS Succinimidyl Ester (ThermoFisher: A37573). One vial of Fluor-NHS ester was resuspended in 10 μL dimethyl sulfoxide. 2 μL of the resuspended dye was subsequently added to 100 μg of antibody. The reaction mixture was agitated at 1000 rpm for 1 h at RT using a benchtop mixer (Eppendorf ThermoMixer) before storage at −20 °C until use.

### 2.8 Flow cytometry assay for the screening of ectodomain mutants

Expi293F cells were transfected with plasmid DNA encoding either WT or mutant full-length versions of RVG in a final culture volume of 600 μL. Transfections were conducted using ExpiFectamine as outlined above. After 24 h or 48 h (specified in results), 300 μL of transfected cells were centrifuged at 300 × G for 5 min and resuspended in PBS. Samples were subsequently divided into two before one half was washed in PBS (pH 7.3) and the other in an acidic buffer (50 mM Bis-Tris, 130 mM NaCl, pH 5.8), before being incubated in the same buffers for 20 min at RT. After two washes, cells were stained for 30 min at RT with Alexa Fluor 647-conjugated RVC20 IgG at 2 μg/mL in the same buffer. Staining solutions were removed by washing in pH-matched buffers (as above) twice followed by a final wash in PBS pH 7.3, before running on a BD LSR II cytometer (BD Bioscience). Analysis of flow cytometry data was performed using FlowJo v10.10.0 (BD Bioscience).

For the flow cytometry-based screening of the panel of combinatorial RVG mutants’ expression, the transfection protocol was modified such that the amount of RVG-encoding DNA was 2 % (*w*/w) of that of previous screens (see Fig. S1). The total amount of DNA was maintained by supplementing transfections with irrelevant plasmid DNA.

### 2.9 Production of adenovirus-vectored RVG vaccines

ChAdOx2 RabG and adenoviruses encoding selected wild-type RVG constructs were produced as previously described [29]. Briefly, virus was rescued by transfection of linearised genome into HEK293 Trex cells (ThermoFisher: R71007), followed by growth in the same cells, purification by CsCl density gradient centrifugation and stored in 10 mM Tris, 7.5 % sucrose, 150 mM NaCl, 0.1 % Tween-80, pH 7.8 at −80 °C.

### 2.10 Production of mRNA RVG vaccines

RVG constructs selected for mRNA vaccine production were synthesised by Twist Bioscience, pre-cloned into a pUC57 vector, downstream of a T7 RNA polymerase promoter sequence compatible with co-transcriptional 5′ capping using the CleanCap AG system (TriLink: N-7113), and upstream of a polyadenosine sequence. Each coding sequence was flanked by the same 5′ untranslated region (UTR) used in BNT162b2 (GenBank: PP544446.1) and a 3’ UTR based on partial sequences from the human-derived mitochondrially encoded 12S rRNA and amino acid enhancer of split (AES) transcripts, concatenated head-to-tail as previously described [36]. mRNA synthesis was performed with 100 % substitution of uridine with N1-methylpseudouridine. Size and integrity of the mRNA were confirmed with microcapillary electrophoresis (Tapestation 4200, Agilent).

mRNA constructs were encapsulated using a Nanoassemblr™ Ignite™ instrument (Cytiva) by combining an ethanol-based lipid mix (Genvoy-ILM™, Cytiva) with mRNA diluted in PNI formulation buffer (Cytiva: NWW0043). Ethanol was subsequently removed by buffer exchange to 1× PBS using a 10 kDa MWCO ultracentrifuge filter (Amicon, Millipore). A final clean-up was performed by sterile filtration with 0.2 μm syringe filters (Acrodisc, Cytiva).

All mRNAs were encapsulated with >87 % encapsulation efficiency as measured by a fluorescence-based assay (Ribogreen Quant-it™, ThermoFisher), conducted as per the manufacturer’s recommendations and using RVG mRNA dilutions as a standard. The hydrodynamic size characteristics of the mRNA-LNPs were checked by dynamic light scattering using a Zetasizer Ultra (Malvern Panalytical), revealing a z-average of 93 nm and a polydispersity index of 0.0573 across all constructs. Encapsulated mRNA vaccines were stored at −20 °C in PBS supplemented with 10 % sucrose until use.

### 2.11 Animal studies

Mice were used in accordance with the UK Animals (Scientific Procedures) Act 1986 under project license numbers P9804B4F1 and PP3331087, granted by the UK Home Office. Content of these licences was approved by the local Animal Welfare and Ethical Review Board at the University of Oxford. Experiments were designed and conducted with attention to the 3Rs principles of Replacement, Reduction and Refinement.

Mice were supplied by Inotive, UK. Mice strains and ages are described in the relevant results sections. All vaccines described in this chapter were administered by intramuscular (I.M.) injection under general anaesthesia into quadriceps, in a total volume of 50 μL for ChAdOx2 immunisations and 100 μL for mRNA constructs, divided in halves across the two hind limbs. Animal numbers in each experimental group are outlined in the relevant figure captions.

### 2.12 Virus neutralising antibody titrations

All VNA assays were standardised using the WHO international reference standard.

Mouse serum samples from experiments testing ChAdOx2-vectored RVG constructs were measured by Rapid Fluorescent Focus Inhibition Test at the Wistar Institute, USA, using heat-inactivated serum samples [37].

Samples from mRNA-vaccinated mice were measured by Fluorescent Antibody Virus Neutralisation assay [38] conducted by BioBest Laboratories UK. Serum samples tested at BioBest were not heat-inactivated prior to testing.

## 3 Results

### 3.1 Characterisation of wild-type RVG constructs in vitro and in adenovirus vectors

**Fig. 1:**
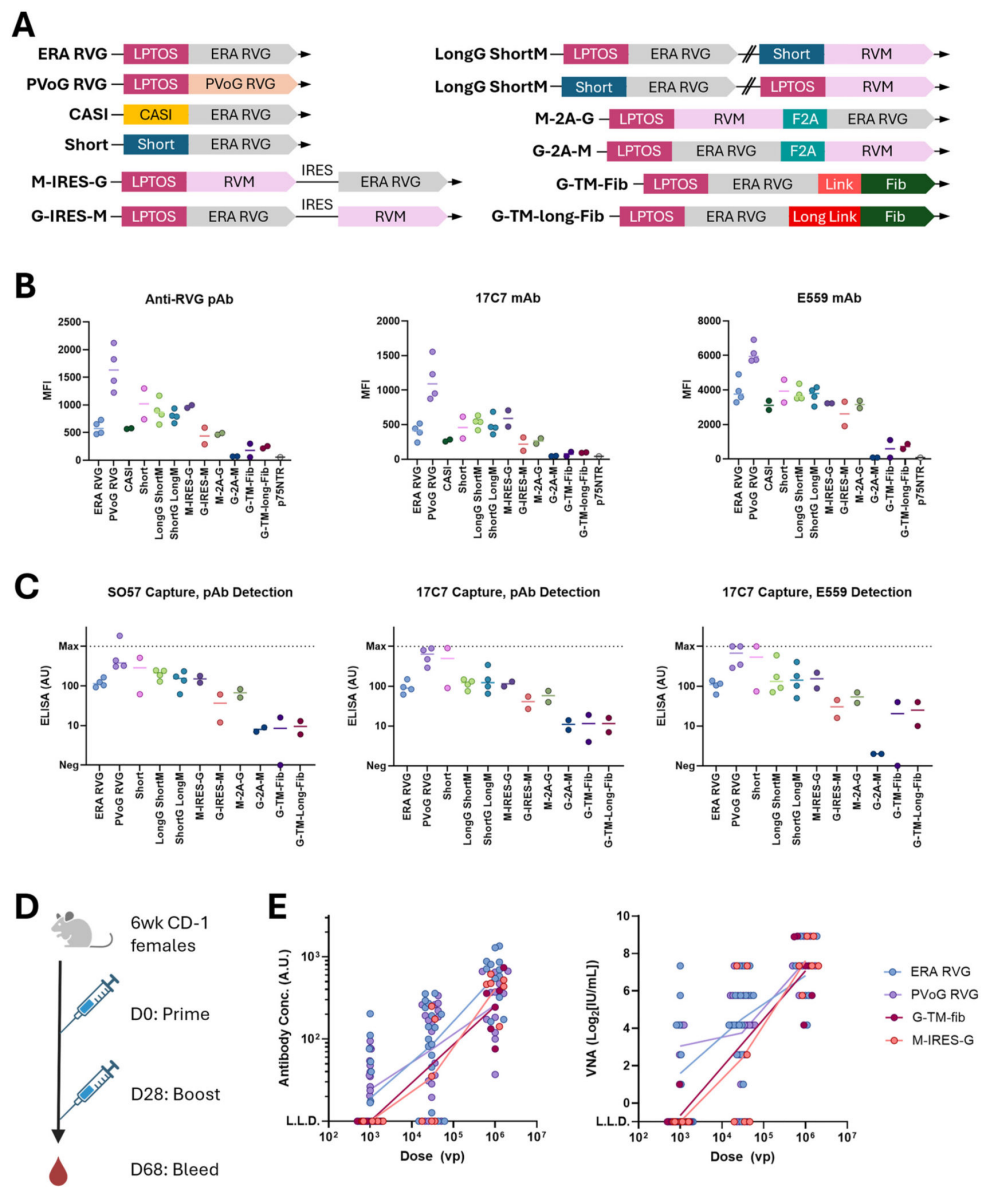
Characterisation of wild-type RVG expression cassettes. We pursued multiple strategies including comparing different gene promoters, modifying the RVG antigen to encourage trimerisation (similar to a strategy described previously that focused on the RVG ectodomain [39]) and testing expression of RVG and RVM in tandem. (A). DNA constructs are shown in ribbon format. LPTOS, CASI and Short are all promoters derived in part from the CMV immediate early promoter. ERA RVG under the control of the LPTOS promoter is considered the ‘baseline’ construct and is included in the ChAdOx2 rabies vaccine currently in clinical trials [8]. PVoG is a codon-optimised version of the rabies virus glycoprotein [17]. RVM = rabies virus matrix protein. IRES = internal ribosomal entry site. F2A = self-cleaving peptide derived from foot-and-mouth disease virus. Link = GGGSGGG. Long Link = [GGGS]_3_GGG. Fib = Fibritin foldon domain. FibSS = Fibritin foldon with engineered cysteines for inter-protomer disulphide bond formation [40]. Schematic created using BioRender.com. (B). Expression levels of different RVG constructs measured by flow cytometry, 48 h post-transfection. Transfected Expi293F cells were stained with RVG reactive polyclonal serum (left), the 17C7 RVG mAb (centre) and the E559 RVG mAb (right). Cells transfected with plasmid DNA encoding an irrelevant human protein (p75NTR) were used as a negative control. Data points represent individual transfections conducted with two independent DNA preparations. MFI = median fluorescence intensity. Where *n* = 4, duplicate transfections were repeated on a different day. Horizontal lines represent the median of all replicates. (C). Expression levels of the RVG constructs measured by sandwich ELISA. Plates were coated with anti-RVG mAbs SO57 (left) or 17C7 (centre & right). Captured RVG constructs were detected with RVG-reactive polyclonal mouse serum (left & centre) or with the E559 mAb (right). Max = upper limit of interpolation using standard curve. Neg = no detectable signal. CASI was not tested by ELISA given its lack of superiority compared to the other promoters when measured by flow cytometry. For (B) and (C), data points represent individual transfections of Expi293F cells with corresponding plasmid DNA. Transfections were performed in 24-well plates using the 96-well plate transfection method described in the methods but with shaker agitation at 700 rpm and a transfection culture volume of 5 mL. (D). Immunisation regimen for CD-1 mice administered ChAdOx2-vectored RVG constructs. Immunisations were performed intramuscularly. Schematic created using BioRender.com. (E). Post-boost immune responses to RVG induced by different ChAdOx2-vectored RVG constructs. Total anti-RVG IgG titres measured by ELISA (left) and virus-neutralising antibody titres (right), measured by rapid fluorescent foci inhibition test, across different vaccine doses are shown for each construct. ‘Baseline’ constructs that were considered of high importance for robust comparison (ERA RVG and PVoG RVG PVoG), were tested across three doses (1 × 10^3^, 3 × 10^4^ and 1 × 10^6^ vp/mL) and in larger numbers than the other groups (*n* = 18, n = 18 and *n* = 12 for the three dose groups, respectively). Representative constructs from the different WT ectodomain mutation strategies (G-TM-Fib and M-IRES-G) were tested on fewer mice (*n* = 6 per group) given our prior knowledge of their detrimental effects on RVG expression (See (B) and (C)). For G-TM-fib, only the low (1 × 10^3^ vp) and high (1 × 10^6^ vp) doses were tested. Points represent responses from individual mice. For ERA RVG and PVoG RVG, data is combined across two independent animal experiments. L.L.D. = Lower Limit of Detection.

We evaluated a panel of constructs with the wild-type RVG amino acid sequence, as illustrated in Fig. 1A. Antigen expression was assessed by flow cytometry on intact cells and by ELISA on cell lysates using RVG-reactive polyclonal mouse sera alongside two RVG monoclonal antibodies.

First, three promoters based on the CMV immediate early promoter were compared by assessing expression levels of the WT (ERA strain) RVG sequence. The LPTOS promoter is comprised of the CMV immediate early promoter, the intron A element and a tetracycline operator (tet–O, used to repress antigen expression during adenovirus vector production) [29,41,42]. LPTOS is currently used in existing ChAdOx1 and 2 vectors. The CASI promoter is comprised of the CMV enhancer region, chicken β-actin promoter, and ubiquitin C enhancer region and is substantially shorter than LPTOS, which may be advantageous when incorporating cassettes into viral vectors with size limitations [43]. An additional, shorter version of the tet-O-containing LPTOS promoter, lacking the intron A element was also included, termed ‘Short’ [44]. All promoters achieved similar expression levels of the WT ERA RVG (Fig. 1B, C).

Next, we compared expression of the WT ERA sequence to the codonoptimised PVoG construct, both under the control of the LPTOS promoter. Across both the flow cytometry and ELISA experiments, the PVoG expressed at higher levels than the ERA RVG construct (Fig. 1B, C).

We then investigated the effect of inserting a trimerization domain downstream of the full-length ERA RVG gene (‘RVG-TM’). The T4 fibritin foldon domain (‘Fib’) [45] was attached to the intravirion / intracellular C-terminus of RVG *via* flexible, glycine-rich linkers: either G_3_SG_3_ (‘Link’) or [G_3_S]_3_G_3_ (‘Longlink’). Expression of these constructs was substantially lower than for WT RVG ERA when measured by ELISA or by flow cytometry (Fig. 1B, C).

Finally, we tested six dual-antigen expression constructs encoding RVG and RVM, comprising three configurations of two coding sequences. Each configuration was tested both with RVG in ‘site 1’ and RVM in ‘site 2’, and *vice versa*. First, the two genes were placed under the control of the LPTOS and Short promoters. Second, the genes were placed downstream of a single promoter and separated by an internal ribosomal entry site (IRES) to generate a bicistronic mRNA. Finally, the RVG and RVM coding sequences were separated by a self-cleaving 2 A peptide from foot and mouth disease virus (F2A). Placing the RVG gene under the control of the LPTOS promoter or the Short promoter in the dual-antigen constructs, or separating them using an IRES, did not affect RVG expression compared to the RVG-ERA-only construct under the LPTOS promoter (Fig. 1B, C). The F2A constructs showed decreased expression of RVG, most prominently when the RVM gene was downstream of the cleavage peptide.

We then evaluated *in vivo* immunogenicity of ChAdOx2 vectors delivering selected constructs from each of the approaches described above. Female CD-1 mice were administered two doses of vaccine on day 0 and day 28 before collecting blood samples at day 68 (Fig. 1D). Vaccine responses were compared to the LPTOS WT ERA RVG construct across three different doses, 1 × 10^3^, 3 × 10^4^ and 1 × 10^6^ vp/mL. For both total anti-RVG titres and VNA titres, none of the new vaccine constructs appeared to be more immunogenic than the baseline construct (Fig. 1E), despite the improved antigen expression levels of the PVoG RVG construct *in vitro*.

### 3.2 Targeted mutations within RVG enhance expression and stabilise the pre-fusion conformation

To investigate whether RVG’s expression and pre-fusion stability could be improved by structure-guided mutagenesis, we initially designed candidate mutations using a homology model based upon the pre-fusion structure of vesicular stomatitis virus protein G [46] (see Supplementary Methods). 72 mutations were constructed from the PVoG version of the WT RVG sequence, using seven mutational strategies (Table 1, Fig. S2).

**Fig. 2:**
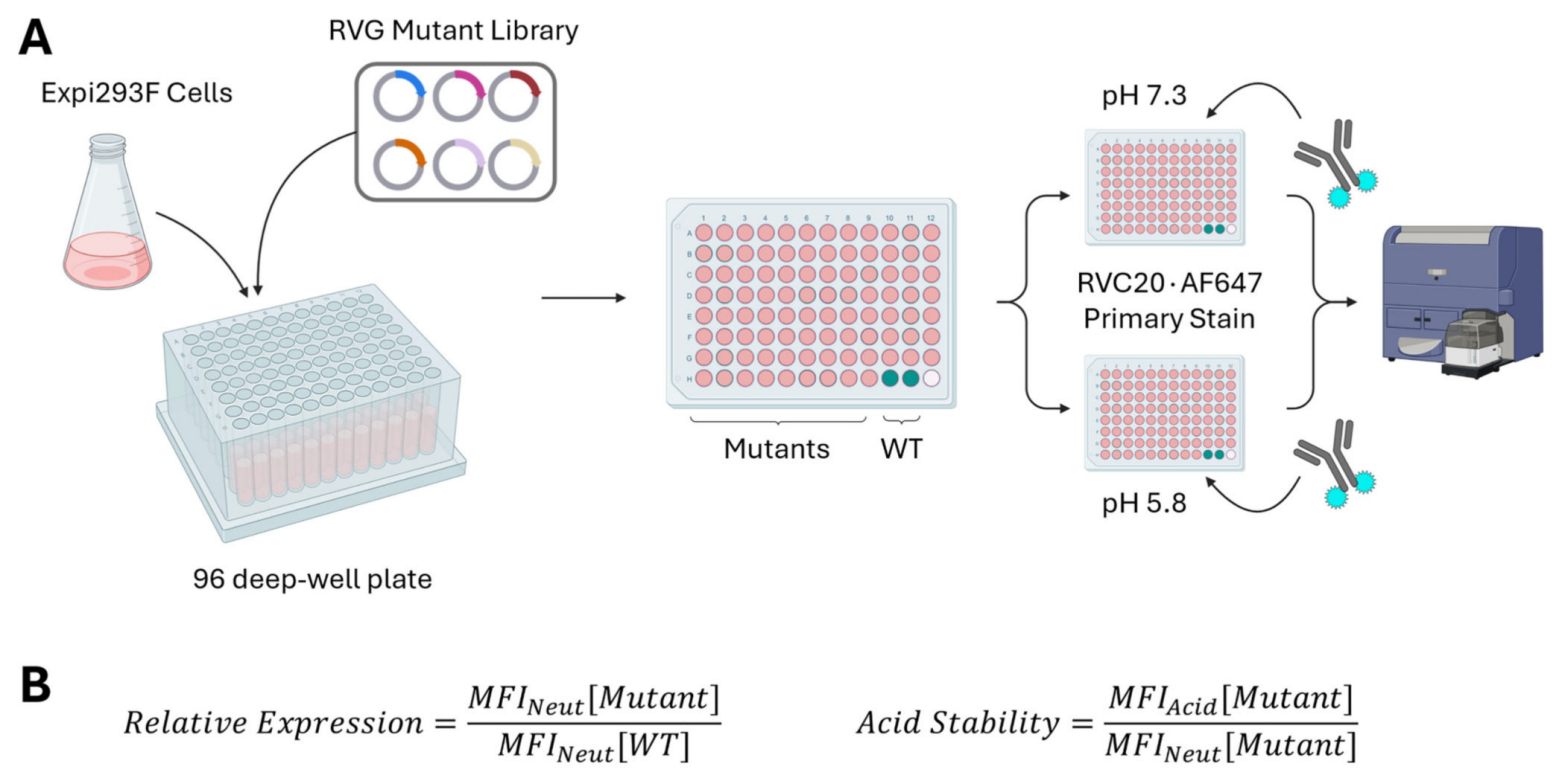
96 deep-well plate-based transient transfections of the RVG mutant library. (A). For each experimental campaign, mutants were transfected in duplicate using independent preps of plasmid DNA. To avoid position effects in the 96-well plate, the order of the mutants on each plate was randomised. The entire assay was performed twice on different days. Staining was conducted with a fluorophore-conjugated RVC20 antibody at two different pHs. Schematic created using BioRender.com. (B). Calculations for relative expression scores (mutant to the WT) and each mutant’s acid stability score. MFI = median fluorescence intensity for stained cell populations transfected with a RVG construct. An ideal mutant would have a relative expression score > > 1, and an acid stability ratio approaching 1.

We aimed to identify mutants of RVG that improved expression relative to the WT and/or stabilised its pre-fusion conformation. A medium-throughput transient transfection assay was developed in which Expi293F cells (ThermoFisher) were transfected with plasmid DNA encoding either the WT RVG (PVoG) or a mutant version and subsequently stained with a pre-fusion conformation-specific mAb, RVC20 [16], conjugated to AlexaFluor 647 (Fig. 2).

Staining was performed at both pH 7.3 and pH 5.8. Median fluorescence intensity at pH 7.3 (MFI_Neut_) was used as a marker for expression level, normalised to MFI_Neut_ of WT protein. The ratio of staining at pH 5.8 (MFI_Acid_) divided by that at pH 7.3 (MFI_Neut_) was used as a marker of pre-fusion stability. WT RVG is known to adopt the post-fusion conformation at pH 5.8 and below (*e.g*. during endosomal acidification) and hence was expected to have a low MFI_Acid_:MFI_Neut_ ratio; stabilisation in the pre-fusion form would be reflected by MFI_Acid_:MFI-_Neut_ approaching 1.

**Fig. 3:**
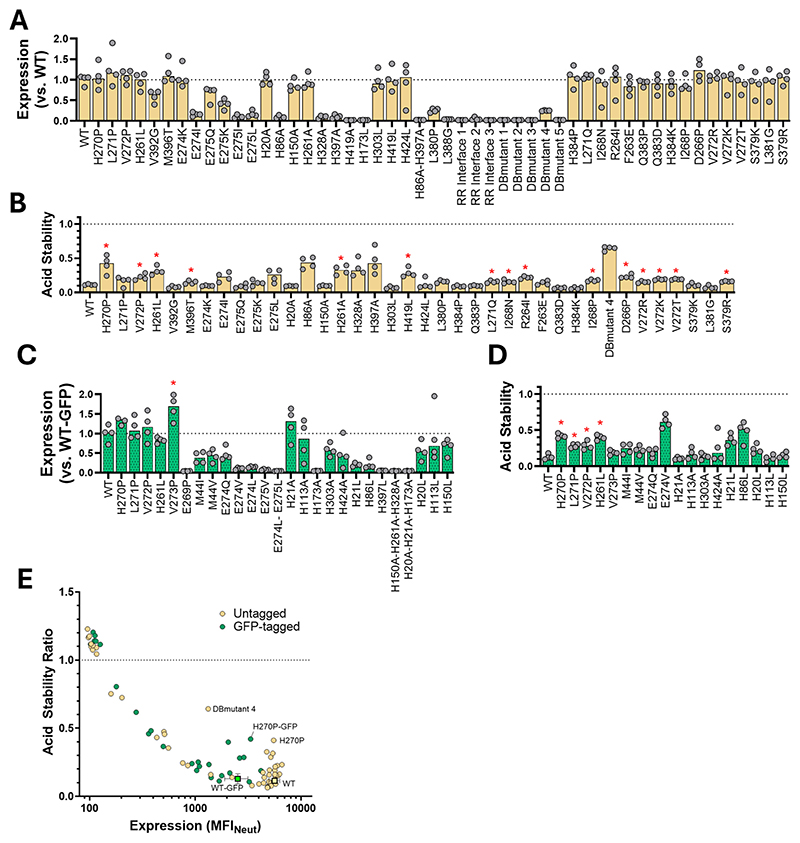
*in vitro* screening of RVG single mutants. (A). Expression levels for each RVG variant 48 h post-transfection, determined *via* their MFI_Neut_ after staining with RVC20 conjugated to AlexaFluor 647. Values are reported as fold-changes relative to the WT RVG, with the average of the WT replicates set to an expression value of 1. Data points represent an individual replicate transfection with data pooled across experiments on different days. Bars display the median of the technical replicates. Mutants labelled ‘RR Interface…’ correspond to mutants in the Rosetta redesign of the trimeric interface group. Mutants labelled ‘DB mutant…’ correspond to cysteine-incorporating mutants intended to introduce stabilising disulphide bonds. (B). Acid stability ratios reported for all mutants that did not completely abrogate expression in panel (A). Stability ratios were calculated as per the method in Fig. 2. Data points represent an individual replicate transfection with data pooled across experiments on different days. Bars display the median of the technical replicates. Red stars indicate mutants that exhibit >70 % expression of the WT and acid stability scores significantly higher than the WT (determined using a false discovery approach) (C). Expression levels for each GFP-tagged RVG variant, calculated and reported as in (A) with the modification that mutant expression is displayed as relative expression to the GFP-tagged WT RVG. Data points represent an individual replicate transfection with data pooled across experiments on different days. Bars display the median of the technical replicates. V273P (starred) was the only mutant identified as significantly enhancing expression (calculated as in (B)). (D). Acid stability ratios reported for all GFP-tagged mutants, calculated and displayed as in panel (B). Red stars indicate significant enhancement of acid stability, as in (B). (E). XY plot displaying acid stability ratios against MFI_Neut_ (expression) values for each RVG variant including GFP-tagged (green), untagged (yellow) and their corresponding WT comparators (squares). Data points represent average values across all technical replicates. For clarity, error bars representing the standard error for each mutant are only displayed for WT constructs. (For interpretation of the references to colour in this figure legend, the reader is referred to the web version of this article.)

22 mutants were constructed with a GFP tag (originally with a view to screening by fluorescence size exclusion chromatography [54]), and were compared to GFP-tagged WT protein. 47 were untagged and were compared to untagged WT protein, and an additional 4 were made both tagged and untagged. Many mutations essentially abrogated expression (Fig. 3A, C). This was common for mutants that targeted the monomer interface *via* redesign in Rosetta (termed ‘RR Interface…’ in Fig. 3), as well as mutations that introduce pre-fusion-locking disulphide bonds (termed ‘DB Mutant…’ in Fig. 3). Mutants that modified the hinge region between L261 and E274 were generally well-tolerated, as were modifications of histidine to leucine (but usually not to alanine).

33 mutants in the panel exhibited expression levels >70 % of the WT (matching to GFP-tagged or untagged WT) (Fig. 3A, C), with one mutant, V273P-GFP, displaying statistically significant enhancement of expression (false-discovery rate [FDR] <5 %, see Fig. S3). Of the constructs with expression levels >70 % of WT, 20 showed a > 2-fold improvement to the acid stability ratio (Fig. 3 B, D). No constructs displayed statistically significant enhancement of both expression and acid stability (again using FDR < 5 %).

Across both expression levels and acid stability, and indeed in both its GFP-tagged and untagged versions, the H270P helix-breaking mutation appeared to be the most promising single mutant (Fig. 5E). This finding was exploited in our previously reported work, in which we used a H270P mutant protein to obtain a structure of RVG’s pre-fusion conformation [47].

**Fig. 4:**
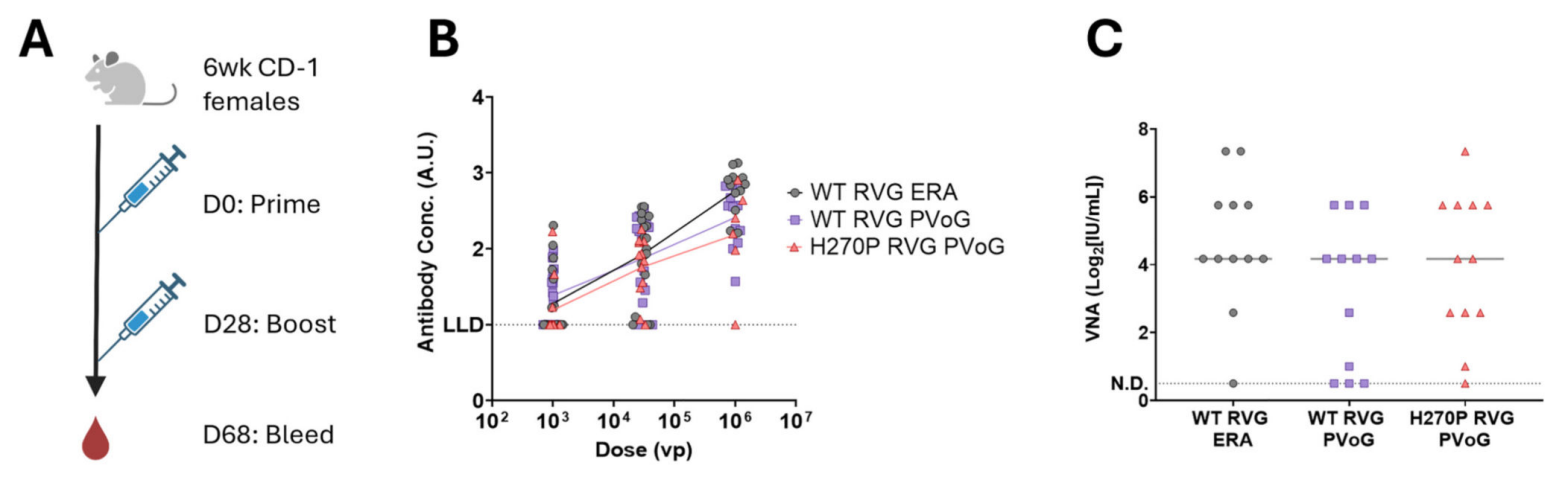
*in vivo* screening of the ChAdOx2-vectored H270P RVG mutant. (A). Immunisation and blood sampling schedule for ChAdOx2 RVG-immunised CD-1 mice. Schematic created using BioRender.com. (B). Post-boost total IgG responses to the WT RVG antigen, as measured by ELISA, raised against different doses of the ChAdOx2 RVG vaccines. Points represent individual mice. Data for the ‘baseline’ constructs WT RVG ERA and WT RVG PVoG are the same as those in Fig. 1 across low-, medium- and high- doses (n = 18, 18, 12, respectively, from two independent animal experiments). The H270P RVG PVoG vaccine was constructed later and tested in a single experiment over the same doses (n = 12, 12, 6 per group). Lines connect the mean response at each dose group for each vaccine construct. LLD = Lower limit of detection. (C). Post-boost virus neutralising antibody titres for mouse samples from the middle dose group in (B) (3 × 10^4^ vp). Points represent individual mice. Horizontal grey lines represent the median response. N.D. = none detected.

The H270P was therefore selected for immunogenicity testing in mice using the same ChAdOx2 vector and regimen as described earlier. Comparison of the H270P construct to its PVoG WT parent, as well as the original WT ERA sequence, revealed no differences in either the total anti-RVG titres raised to the vaccine across three tested doses, nor the VNA titres for animals immunised with 3 × 10^4^ vp/mL (Fig. 4).

### 3.3 Combining mutants of interest further improves RVG’s acid stability, but does not affect immunogenicity of mRNA vaccines compared to the WT

Using a cut-off expression value of >70 % WT expression, 10 prefusion stabilising mutations from the untagged panel (H270P, H261L, R264I, D266P, I268N, L271Q, V272P, S379R, M396T, H419L) and one mutant from the GFP-tagged panel (V273P) were selected to construct 43 double-mutants and 3 triple-mutants. These mutants, summarised in Table S1, were investigated to determine if their effects on RVG were additive.

**Fig. 5:**
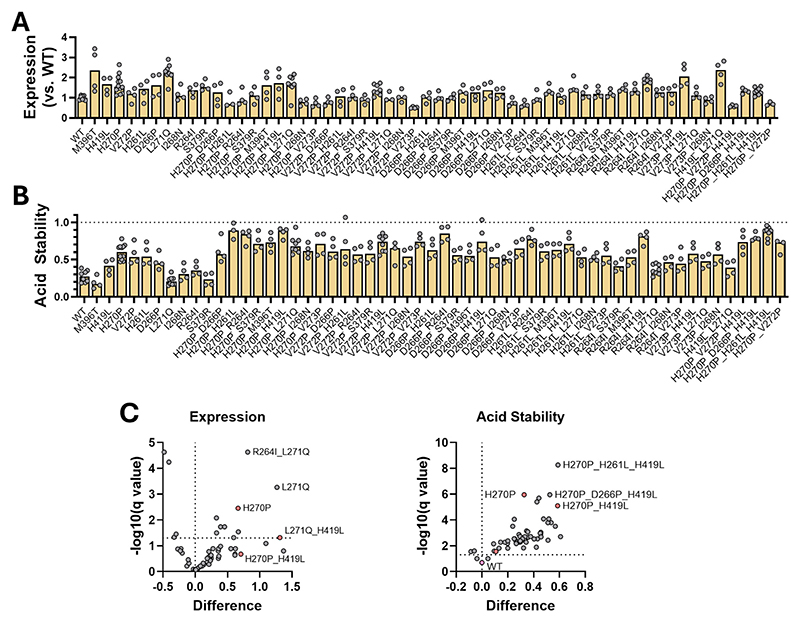
*in vitro* screening of RVG combinatorial mutants. (A). Expression levels of RVG double mutants and their single-mutant parents, 24 h post-transfection. Values are reported as fold-changes relative to the WT RVG, with the average of the WT replicates set to an expression value of 1. Data points represent technical replicates. Bars represent median values for each mutant. All transfections performed with 2 % the original amount of RVG DNA as in Fig. 3 to improve that chances of detecting differences in antigen expression levels (see Fig. S1). (B). Acid stability ratios of double mutants and their single-mutant parents. Data points and bars as in (A). (C). Volcano plots displaying the effects of mutation on expression relative to the WT (left), and acid stability (right). Statistical significance was determined using a false discovery approach (FDR < 5 %). Coloured data points represent constructs selected for *in vivo* testing as mRNA vaccines.

Combinatorial mutants were screened alongside their single-mutant parents. At this point we also performed further optimisation of the expression and stability assay (Fig. S1). Although combining individually beneficial mutations did not always produce a compound effect, a number of double-mutants containing the L271Q mutation exhibited enhanced expression (Fig. 5A). The acid-stabilising effects of individual mutations were additive for several mutant pairs. Six double-mutants exhibited acid stability ratios >0.8, an improvement upon the best individual mutant (H270P, ratio 0.5) (Fig. 5B). Mutant L271Q_H419L exhibited the greatest improvement to antigen expression, while the H270P_H419L combination exhibited the joint-highest acid stability score with H261L_H270P_H419L and H261L_H270P (Fig. 5C).

L271Q_H419L and H270P_H419L were selected for *in vivo* immunogenicity evaluation, in this case using lipid nanoparticle-encapsulated mRNA constructs. We speculated that RNA vaccines may be more sensitive to beneficial effects of mutations enhancing antigen output per mRNA molecule than would be the case for adenovirus-vectored vaccines (in which a strong promoter results in multiple transcripts in each cell, potentially causing saturating expression).

We selected an mRNA dose of 0.2 μg, on the steepest part of the dose-response curve (Fig. S4), to maximise the chances of observing any differences in induced antibody titres between mutants and the WT.

**Fig. 6:**
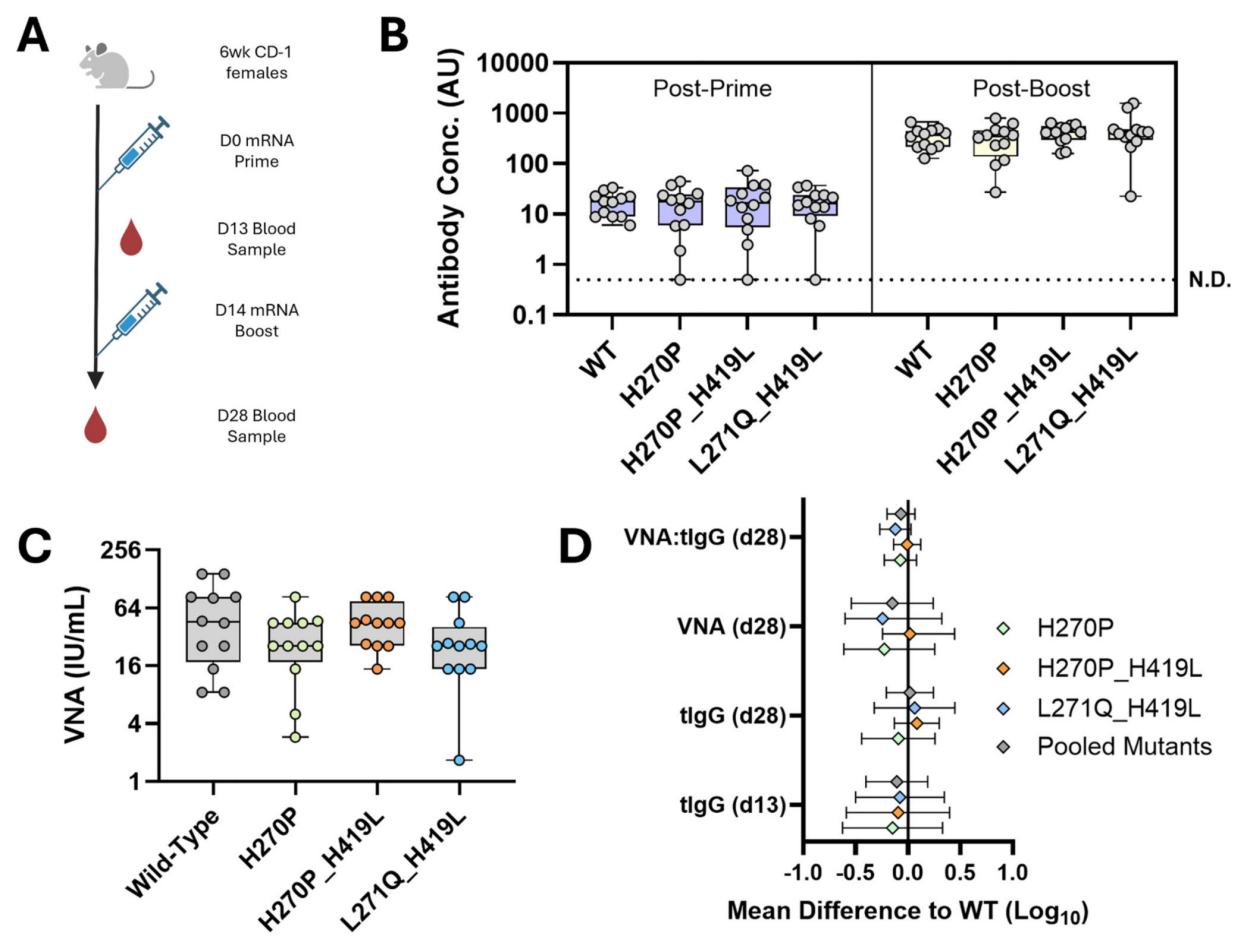
Immune responses to mutant RVG mRNA vaccines. (A). Immunisation and blood sampling schedule for RVG mRNA-immunised CD-1 mice. Prime and boosts were 0.2 μg/dose mRNA. All RVG mRNA constructs were tested at n = 12 per group. Schematic created using BioRender.com. (B). Post-prime and post-boost total IgG responses to WT RVG, as measured by ELISA, for mice immunised with different RVG mutant mRNA vaccines. N.D. = No detectable response (C). Virus neutralising antibody titres determined for the same mouse samples in (B) at the post-boost timepoint. (D). For (B) and (C) data points represent individual mouse responses, boxes represent the inter-quartile range, error bars display the response range and horizontal black lines show the median for each group. (E). Differences in the mean response values for three mRNA vaccines encoding variants of RVG, relative to the WT mRNA construct. For each immunological readout (Y-axis), geometric means were compared to the WT and differences plotted (diamonds). A mean difference of 0 represents no change from the WT response. Positive mean differences correspond to higher induced titres than the WT. Error bars represent the 95 % confidence intervals for each RVG mutant and for each immunological readout, all of which show overlap with x=0, suggesting no significant differences between the WT and any mutant RVG vaccine in any readout. In all cases, mean differences and confidence intervals were calculated by a one-way Brown-Forsythe and Welch ANOVA with Dunnett’s T3 multiple comparisons test. Statistical analysis was performed using GraphPad Prism. (For interpretation of the references to colour in this figure legend, the reader is referred to the web version of this article.)

The two double mutants were compared to the parent WT PVoG sequence and the H270P single mutant. The latter was included given it was the most stabilising single mutant identified and to permit comparison to the work of Cao et al. [55]. Female CD-1 mice were administered 2 doses of an LNP-encapsulated mRNA vaccine encoding the WT RVG or a mutant construct (Fig. 6A). We subsequently conducted total anti-RVG IgG (tIgG hereon) ELISAs post-prime and post-boost (Fig. 6B) and assessed VNA titres after the boost (Fig. 6C).

None of the mutant constructs induced higher anti-RVG tIgG or VNA antibody responses than the WT. To investigate whether the mutants might have a subtle beneficial effect upon antibody quality, we also calculated post-boost VNA:tIgG ratios for animals vaccinated with each immunogen. We calculated mean differences between each mutant and the WT for each parameter and 95 % confidence intervals for these differences (Fig. 6D). We conclude that the mutations we identified as improving RVG expression and/or pre-fusion stabilisation *in vitro* do not convey any immunological advantage in mice using an mRNA vaccine platform.

## 4 Discussion

Improved rabies vaccines may reduce rabies mortality. Here, we sought to engineer RVG, the target of protective neutralising antibodies, to improve its expression and/or stability in the pre-fusion conformation, which may facilitate development of next-generation vaccines. Whilst VNA alone may not fully predict a vaccine’s effectiveness in providing protection against rabies, the well-established VNA threshold for vaccine induced protection makes it a useful focal point for optimising new vaccine constructs and regimens, particularly when screening multiple constructs.

First, we investigated whether codon optimisation, different promoters, co-expression with matrix protein or trimerisation domains might improve RVG expression relative to a WT comparator. Though WT-equivalent or higher expression levels were achieved for a subset of these constructs, exemplars tested in the form of ChAdOx2 vaccines in mice did not exhibit improved immunogenicity.

We then focused on design and evaluation of RVG mutants, exploring seven different strategies (Table 1). Of the 72 ‘single mutants’ screened initially, several mutations appeared to stabilise the pre-fusion conformation, with many of the promising mutations targeting residues 261–273 which undergo rearrangement during conformational transition. Eleven promising individual mutations (designed using four of the strategies) were used to design a second set of combinatorial mutants.

We had previously described the H270P mutation, which was the most stabilising single mutation and had facilitated our elucidation of the pre-fusion RVG structure by cryo-electron microscopy [47]. Combining H270P with a putative histidine switch-targeting mutation, H419L, located just prior to the transmembrane domain resulted in further pre-fusion stabilisation (Fig. 6). Given that H419 is not resolved in any pre- or post-fusion RVG structures [16,47,49,56], it may not form part of any rigid secondary structure elements. Instead, mutating H419L may abrogate RVG’s ability to detect the drop in pH that triggers the pre- to post-fusion transition in the WT [57,58]. We would speculate that the greater acid stability observed by combining the H270P and H419L mutations may be the result of two orthogonal stabilisation mechanisms operating in tandem.

Several previous reports have described encouraging pre-clinical immunogenicity of mRNA rabies vaccines [55,59–61]. Notably, one report published since our original description of the stabilising effect of the H270P mutation suggested a ~ 2.3-fold enhancement of VNA induction by RNA vaccination of mice using an H270P construct, as compared to WT [55]. In contrast, we found that the H270P mutation did not convey an immunological advantage when administered as an mRNA vaccine in mice, nor as a ChAdOx2-vectored vaccine. Moreover, pairs of mutations that out-performed H270P with respect to improving acid stability and expression had no detectable effect upon quantitative or qualitative immunogenicity when administered as mRNA vaccines, despite having been administered at a dose level on the steep part of the dose-response curve, and despite use of group sizes providing power to detect relatively modest effects. The consistency of this lack of effect, across two delivery methods and, in the case of the mRNA vaccine studies, three candidates with similar *in vitro* characteristics (two of them including H270P) argues further for our data representing a ‘true negative’ result.

The precise explanation for the discrepancy in these findings is unclear. Whilst this may be related to differences in experimental design (*e. g*. mouse strain, vaccine construct, dose, regimen, immunoassays), it is also possible that improvements conveyed by the H270P mutation on anti-RVG IgG and VNA titres may have been due to chance.

Regarding the differences between the findings of our expression assays and animal experiments, we speculate that, in contrast to the *in vitro* conditions we selected to enable detection of pre-fusion-stabilising and expression-enhancing mutations, *in vivo* conditions in the context of RVG expression from mRNA or viral vector vaccines may tend to attenuate or eliminate any immunological effect of such mutations.

It seems likely that WT RVG, when expressed from an mRNA or viral vector vaccine *in vivo* under physiological conditions (including pH at the external surface of the plasma membrane), spontaneously adopts the pre-fusion conformation. B-cell receptors are most likely to encounter the antigen under such conditions, rather than in acidic compartments where the unstabilised protein would be expected to adopt post-fusion conformation. Our reasoning that vectored delivery of WT RVG is likely to present in the pre-fusion form may go some way to explain why the successes of RSV, MERS and SARS-CoV-2 pre-fusion stabilisation approaches could not be translated to RVG. While these other viral fusion proteins may not be sufficiently stable in the WT to present an immunologically relevant pre-fusion conformation to the immune system, this may not be true of vector-delivered RVG. Additionally, the fusion proteins referenced above all represent Class I fusion proteins, whilst RVG is in Class III; their more distant relationship to RVG may partially explain these differences in observed behaviour.

Moreover, it should be noted that, as is the case for many viral fusion proteins, only a subset of neutralising antibody epitopes are pre-fusion specific (*e.g*. that for RVC20 [16], but not that for 17C7 [47]). If these factors are indeed the explanation for lack of immunological effect of pre-fusion stabilisation seen in this study, it is likely that they would apply also to other mutations which achieve a pre-fusion stabilising effect (unless they also achieve expression enhancement).

With respect to *in vivo* effects of expression enhancement, we found *in vitro* that expression-enhancing effects were difficult to detect unless we selected conditions likely to exhibit sub-maximal RVG expression. Transfecting 2 % RVG-encoding DNA with 98 % irrelevant DNA and assaying expression at 24 h accentuated differences (Fig. S1); transfecting 100 % RVG-encoding DNA and assaying expression at 48 h attenuated differences. If cell surfaces readily become saturated with RVG when supplied with the number of transcripts in a typical LNP or expressed from a viral vector, or if cells lose viability beyond a certain level of RVG expression (for example due to apoptosis, as suggested elsewhere [62]), overcoming this using other constructs designed to enhance expression may have a limited effect. It remains plausible that the expression enhancements we were able to achieve were quantitatively insufficient, and that future constructs achieving greater levels of expression may prove beneficial *in vivo*.

There are varying opinions as to the effect of structure-guided prefusion stabilisation upon immunogenicity of mRNA & viral-vector vaccines. Our data adds further weight to the argument that it should not be assumed that *in vitro* advantages will translate into improved immunogenicity. Effects are likely to vary for different antigens and different delivery systems.

Although we observed a discrepancy between *in vitro* results and *in vivo* performance of mutant-encoding mRNA and adenoviral vaccines, this should not be interpreted as an argument against the use of these stabilising mutations in the production of recombinant RVG protein-based vaccines. For protein vaccines, unlike mRNA and viral vector vaccines, manufacturing yield and stability in storage are determined by characteristics of the protein antigen.

While we set out to design improved RVG transgenes for adenovirus-vectored and mRNA vaccines, our results are cautionary with respect to this approach. It may be that the greatest value of this work is instead in facilitating the production of protein-based rabies vaccines.

## Supplementary Material

Supplementary data to this article can be found online at https://doi.org/10.1016/j.vaccine.2025.127541.

Supplementary 1

Supplementary 2

## Figures and Tables

**Table 1:** Structure-guided mutagenesis strategies for stabilising pre-fusion RVG. We have previously reported preliminary data obtained with two of these seven strategies, indicated by * [47].

Mutation Strategy	Rationale
Disulphide bond Introduction (DBD)	Using an online disulphide engineering tool [48] and a structural model of the pre-fusion RVG designed in Rosetta, cysteine residues were introduced with the aim of introducing disulphide bonds between positions on the RVG that are proximal in the pre-fusion conformation but separated in the post-fusion form, hence preventing the rearrangement of RVG to the post-fusion.
Helix-bend-helix proline introduction*	Residues 269–273 are modelled as a flexible loop in pre-fusion RVG (and unresolved in the basic pH structure of RVG [49]), but form part of an extended α-helix in the post-fusion form. Helix disruption by introducing proline residues might therefore prevent formation of the extended post-fusion helix.
Rosetta Redesign 1: Helix-bend-helix	Using the pre-fusion RVG model, residues 263–272 were re-designed in Rosetta to stabilise the flexible loop.
Histidine switch deactivation*	Acid-dependency of the pre- to post-fusion transition points to an acid sensor in RVG, possibly histidine protonation at acidic pH. Substitution with hydrophobic residues might prevent rearrangement.
Rosetta Redesign 2: residues 376–389	Mutations targeting an unstructured region in the Rosetta pre-fusion RVG model that forms into a helix in the post-fusion structure, with the goal of destabilising the helix.
Rosetta Redesign 3: Pre-fusion trimer interface	The mechanism for the pre- to post-fusion transition is still incompletely understood, but it is thought that the RVG trimer may dissociate to allow for rearrangement [49–52]. Rosetta-guided mutagenesis of the modelled pre-fusion trimer interface aimed to stabilise interactions between monomers to prevent the dissociation of the trimer and therefore the formation of the post-fusion state.
Resistant to Acid-Induced Neutralisation (RAIN)	A set of previously characterised mutants that conveyed resistance to neutralisation by the pre-treatment of virus in acid [53]. The resistance is probably the result of stabilising the high pH, pre-fusion structure of RVG (or a transition intermediate) so that some of the surface RVG trimers remain in the pre-fusion conformation and can still trigger fusion.

## Data Availability

Data will be made available on request.
