# Supplementary material for: Rabies glycoprotein engineering for improved stability and expression
Source: Vaccine. Author manuscript; Available in PMC 2025 Aug 4. (PMC7617983; doi:10.1016/j.vaccine.2025.127541)
Supplement: Supplementary 1 [file EMS207637-supplement-Supplementary_1.docx]

Supplementary Methods, Figures & Tables

# Supplementary Methods

## Molecular biology

DNA strings encoding the WT ectodomain constructs (**Figure 1A**) were ordered from GeneArt (ThermoFisher) and cloned using standard molecular biology techniques into the pENTR4 backbone multiple cloning site as follows. Restriction digests of inserts and backbones were performed using KpnI and NotI for PVoG RVG, ‘Short’ and G-2A-M, constructs encoding the fibritin trimerisation domain and for constructs for the tandem expression of RVG and RVM. The CASI construct was cloned using KpnI and HindIII. The M-IRES-G and M-2A-G constructs were produced by InFusion after pre-digesting insert and backbone with HindIII. G-IRES-M was made equivalently using NotI. All restriction enzymes were supplied by New England Biolabs.

## ELISA

Wells of the ELISA plates were coated with 17C7 or SO57 overnight at 4 °C. The following morning, ELISA plates were washed 6 times in PBS, 0.05% Tween-20 (Merck: P9416, PBS/T hereon) and blocked with blocker casein (ThermoFisher: 37528) for 1 h at room temperature (RT), 200 μL/well. After removing blocking agent, transfected cell lysates (diluted 1:50 in 50 mM HEPES pH 7.4, 150 mM NaCl, 1.2% β-OG) were added to wells of the plate and incubated for 1 h at RT. Plates were washed 6 times, as above.

Staining was performed using either a 1:5000 dilution in PBS of RVG-reactive polyclonal mouse serum pooled from animals immunised with an inactivated rabies virus (IRV) vaccine (Rabipur, Valneva), or with the E559 anti-RVG mAb (Site II) [1] at 2 μg/mL, and left at RT for 1h. Finally, wells of the ELISA plate were stained with a goat anti-mouse IgG Fc-specific secondary antibody conjugated to alkaline phosphatase (Merck: A3562) at a dilution of 1:1000 for 1 h at RT.

Plates were developed using 20 mg 4-nitrophenyl phosphate tablets (Merck: N2765) and diethanolamine buffer (ThermoFisher: 34064), formulated as per the manufacturers’ instructions, 100 μL/well. Each plate was allowed to develop for 10-15 min before reading on a CLARIOstar Plus Microplate Reader (BMG Labtech).

Raw OD_405 nm_ values were converted into Arbitrary Units (A.U.) by interpolation using a standard curve present on every ELISA plate. Standard curves were generated from a 10-point, 2-fold dilution series made with a positive control sample of full-length RVG C-tag purified by the method described above, starting at 3.7 μg/mL.

## Design of RVG mutants for pre-fusion stabilisation

RabG pre-fusion and post-fusion models were generated using the I-TASSER server. To prepare these models for design calculations using the Rosetta scoring function, they were subjected to structural refinement with the Rosetta FastRelax protocol. Following relaxation, the models were manually inspected to identify key residues that might play a role in the conformational transition or in stabilizing either the pre-fusion or post-fusion state (see Table 1). Residues were selected based on their structural location, flexibility, involvement in inter- or intra-domain interactions. A curated list of candidate residues was then subjected to *in silico* mutational design using the Rosetta FastRelax protocol with design enabled. The designed residues were carefully analysed in the context of both the pre-fusion and post-fusion models. Comparative structural assessments were performed to evaluate how the mutations might alter conformational preferences. From this analysis, a subset of mutations (see **Supplementary Table S1**), predicted to either destabilize the post-fusion state or stabilize the pre-fusion state, was selected for experimental validation.

## Transient Transfection of RVG mRNA

3 ×10^5^ HEK293T cells were transfected with 1 μg unencapsulated WT RVG mRNA in a 6-well tissue culture plate using the mRNAfect transfection reagent (Tebubio: 80-40). Complexes were prepared using a 5:1 ratio of mRNAfect:mRNA (μL:μg), using a GFP mRNA transfection condition as a positive control following the same protocol. RVG mRNA-transfected cells were harvested at the 24 h and 48 h timepoints and stained for flow cytometry under neutral conditions by the same method described above for the in vitro screening assay.

## NanoGlo Luciferase Transfection Reporter Readout

To assess the efficiency of Expi293F cell transient transfections, a NanoLuc luciferase (Promega, GenBank ID: JQ513378.1) reporter plasmid was spiked into each transfection at 0.05% of the total plasmid DNA mass. Luciferase readouts were determined by adding 100 μL of the development mix (2 mL PBS, 100 μL NanoGlo assay buffer, 1 μL NanoGlo substrate, Promega: N1110) to 2 μL of each transfection supernatant and immediately measuring luminescence after excitation at λ_max_ = 460 nm using a ClarioStar Plus Microplate Reader (BMG Labtech). In general, luciferase activity readouts were used simply as a sense-check for successful transfection. For transfections in flasks, scores >1 ×10^5^ relative luciferase units (RLU) were considered high efficiency. Scores <5 ×10^3^ RLU were considered low efficiency, suggesting erroneous transfection and were excluded from analyses. Luciferase scores in plate-based transfections were generally 10-fold lower than those of flasks.

## Statistical Analyses

Statistical tests were conducted using GraphPad Prism version 10.4.

Comparison of each RVG mutant’s expression and pre-fusion stability to the WT were made using by multiple unpaired T-tests with Welch’s correction. Multiple comparison was adjusted for by implementing a false discovery rate of 5% using the Benjamini, Krieger and Yekutieli method.

To compare mutant RVG constructs to the WT across different immunological readouts from the animal experiments, responses induced by each mutant construct were compared to the WT by one-way Brown-Forsythe and Welch ANOVA with Dunnett’s T3 multiple comparison test. Comparisons were made using the mean differences for each mutant relative to the WT.

## Animal Studies

In the design of the mRNA vaccine comparison experiment, a power calculation was performed to determine the size of each experimental group (one-way ANOVA, α=0.05, β=0.8). Calculations were performed in GPower 3.1 using the variance observed in the 0.15 μg dose-group from the WT RVG mRNA vaccine pilot experiment (**Figure S4**). The predicted effect size (increase in ELISA or VNA response in animals receiving variant vaccines, as compared to the WT) was 0.51 log_10_-fold, in line with a recently published RVG mouse study [2].

# Supplementary Figures

## Figure S1


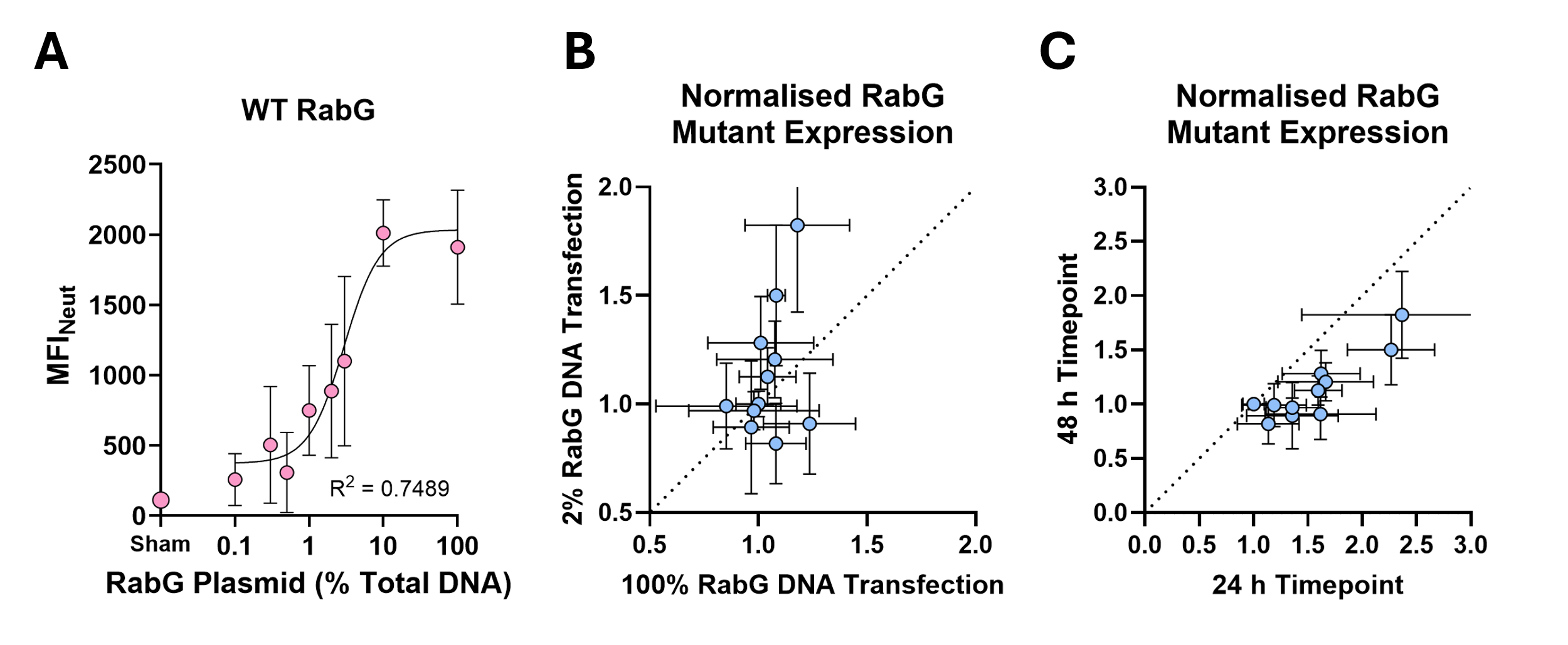
***Figure S 1: Refinement of the RVG in vitro expression assay.***

1. XY plot displaying RVG antigen expression, measured using the median fluorescence intensity of transfected cells stained with the RVC20 mAb conjugated to AlexaFluor647, as a function of the amount of RVG DNA used in the transient transfection. 100% RVG Plasmid corresponds to a DNA concentration of 1 μg/mL transfected cells and corresponds to antigen expression levels that above the steep part of the dose-response curve. The sham condition used 100% non-RVG plasmid DNA (see methods). Data points represent the average MFI_Neut_ values across all technical replicates (n = 6 per dose). Where NanoGlo luciferase values indicated a failure of transient transfection for any replicates, data was excluded from the analysis. These exclusions led to smaller sample sizes for the 0.5% condition (n = 3) and 1% condition (n = 5). The plotted line and corresponding R^2^ value represent a 4-parameter sigmoidal curve fitted to the log-transformed DNA dose data. Curve fitting was conducted in GraphPad Prism. For screening combinatorial RVG mutants, 2% the original RVG-encoding plasmid DNA concentration was selected to improve the chances of detecting differences in antigen expression between mutants and the WT.
2. XY plot displaying the expression levels of the 10 single, untagged mutants selected for design of combinatorial double mutants (see **Figure 6**) transfected using 100% RVG DNA and 2% RVG DNA. Relative expression values displayed were calculated as described in (**Figure 3B**) comparing to WT RVG transfections using 100% and 2% RVG DNA correspondingly. Data points represent average values across technical replicates for each mutant (n = 4 for all variants except WT, L271Q, H270P_L271Q, V272P_H419L, R264I_L271Q, H270P_H261L_H419L where n = 8, and H270P where n = 12). Error bars represent the standard deviation calculated across technical replicates. Dotted line represents 𝑦=𝑥.
3. XY plot displaying the relative expression levels of mutants in (B) sampled at 24 h and 48 h post transfection with 2% RVG DNA. Data points represent average values across technical replicates for each mutant (replicates as per (B)). Error bars represent the standard deviation calculated across technical replicates. Dotted line represents 𝑦=𝑥.

## Figure S2


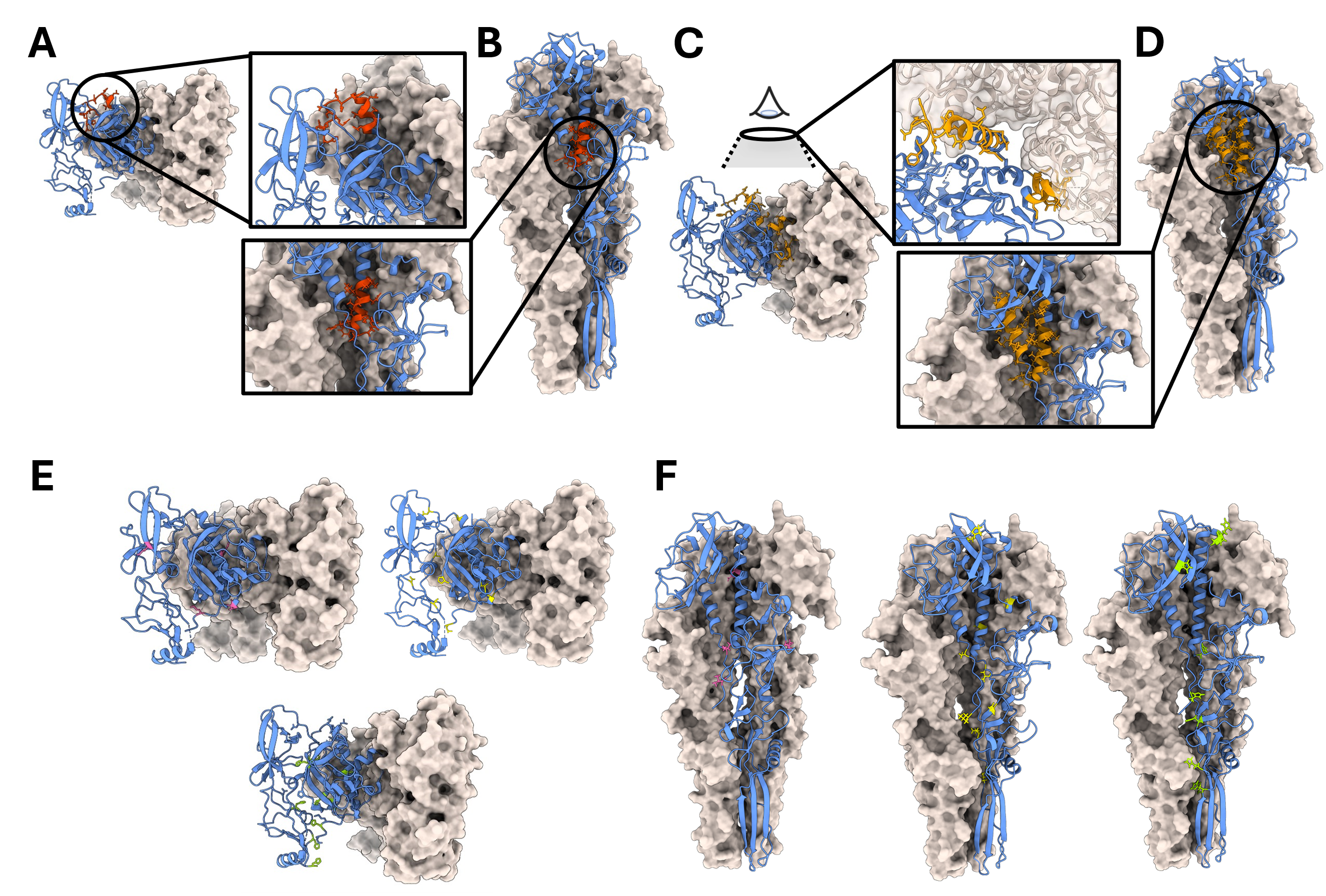


**Figure S 2: Structure-guided RVG mutants.**

Trimeric RVG ectodomain in the pre-fusion (PDB: 8A1E) and post-fusion conformations (PDB: 6LGW). For all structures, a single monomer is shown in ribbon format (blue), and the remaining monomers as surfaces (grey). All structures were visualised in ChimeraX [3].

1. Helix-bend-helix mutations (red) on pre-fusion RVG with the top of the trimer highlighted on the inset.
2. Helix-bend-helix mutations (red) on post-fusion RVG with the newly formed extended α-helix highlighted on the inset.
3. Regions of the pre-fusion RVG redesigned computationally using Rosetta (orange). A top-down view of the trimeric interface is highlighted on the inset.
4. The same Rosetta-redesigned residues highlighted on the post-fusion RVG structure.
5. The pre-fusion RVG structure highlight residues mutated made under the RAIN strategy (upper left, pink), disulphide by design strategy (upper right, yellow) and histidine-switch strategy (bottom, lime), respectively.
6. The same mutation strategies in (E) displayed on the post-fusion RVG structure.

## Figure S3


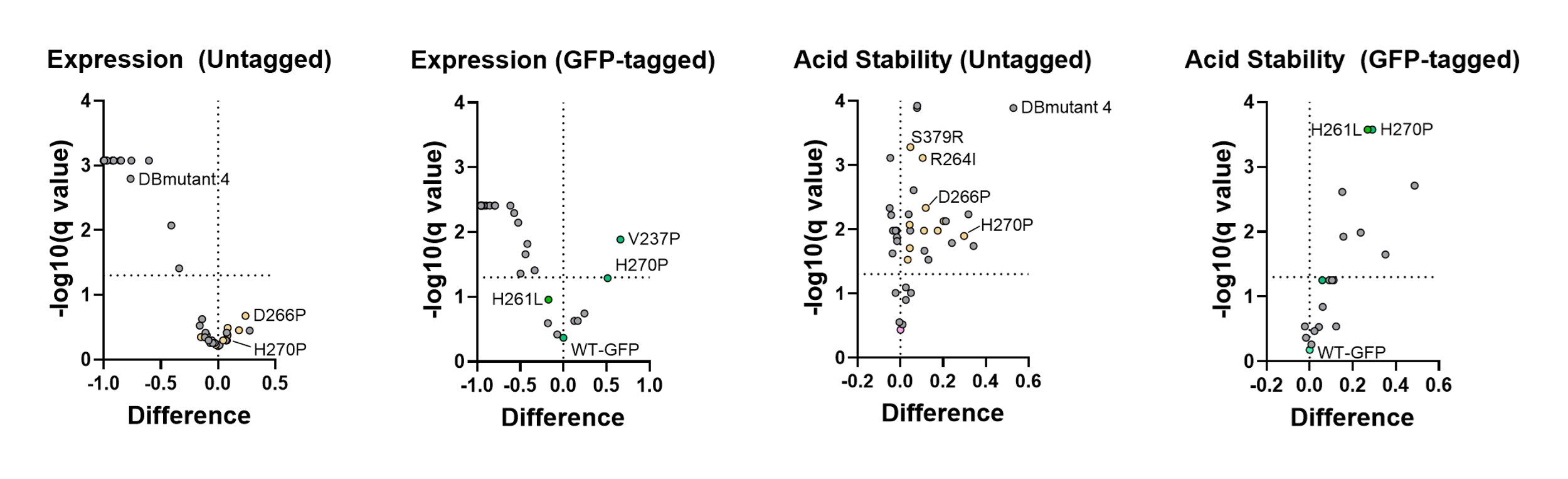


**Figure S 3: Volcano plots displaying each RVG mutant’s effect on expression and the pre-fusion stability under acidic conditions.** Plots are shown for untagged and GFP-tagged constructs. Data points represent individual RVG variants. In all cases, expression and acid stability levels were compared to the WT (or WT-GFP) by multiple unpaired t-tests with Welch’s correction, assuming individual variance for each mutant and implementing a false discovery rate of 5% using the two-stage step-up method developed by Benjamini, Krieger and Yekutieli. Differences are plotted such that positive values correspond to greater expression and acid stability in relevant graphs. Horizontal dotted lines represent a significance cut-off of p<0.05. Coloured circles represent mutants that were selected to construct combinatorial mutants. Statistical tests were conducted using GraphPad Prism.

## Figure S4


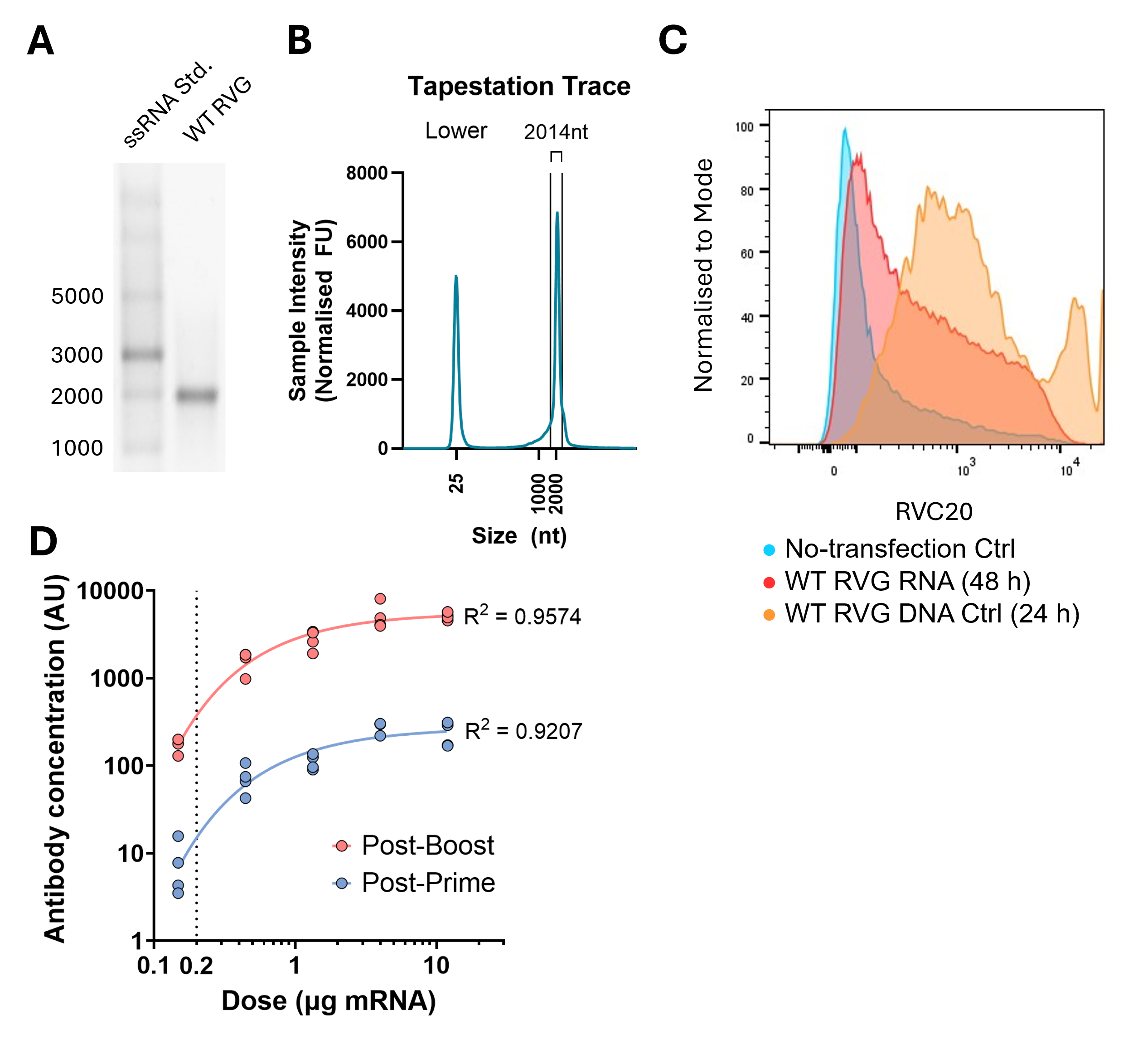


**Figure S 4: WT RVG mRNA vaccine production and screening.**

1. RNA agarose gel of WT RVG mRNA vaccine construct produced by in vitro transcription with ssRNA ladder (NEB: N0362S).
2. Tapestation trace for WT RVG mRNA produced by in vitro transcription, re-graphed in GraphPad Prism. ‘Lower’ peak represents Tapestation 25 nt standard peak. Median transcript size for WT RVG construct calculated automatically on Tapestation (Agilent Technologies).
3. Histogram representing fluorescence intensity in 670/30 filter after staining transfected cells with RVC20 mAb conjugated to AlexaFluor 647. Positive control cells (orange) were transfected with plasmid 100% RVG DNA as described in the methods.
4. Post-prime (blue) and post-boost (red) total IgG responses to WT RVG, as measured by ELISA, plotted as a function of mRNA vaccine dose. Data points represent individual mouse responses. Dose response curves were determined by fitting of a 4-parameter curve to the doubly log-transformed data, with corresponding R^2^ values for each curve also calculated. Curve analysis was performed using GraphPad Prism. The dotted vertical line demarcates the vaccine dose selected for further mouse experiments.

# Supplementary Tables

## Table S1

| **Mutation Strategy** | **Mutation (pseudonym)** |
| --- | --- |
| Disulphide bond Introduction (DBD) | P13C + V139C (DBmutant 1) |
|  | D32C + H270C (DBmutant 2) |
|  | I134C + L399C (DBmutant 3) |
|  | D143C + L260C (DBmutant 4) |
|  | V296C + S391C (DBmutant 5) |
| Helix-bend-helix proline introduction | E269P |
|  | H270P |
|  | L271P |
|  | V272P |
|  | V273P |
| Rosetta Redesign 1: Helix-bend-helix | F263E |
|  | R264I |
|  | R264E* |
|  | D266P |
|  | I268N |
|  | I268P |
|  | L271Q |
|  | V272K |
|  | V272R |
|  | V272T |
| Histidine switch deactivation | H20A |
|  | H20L |
|  | H21A |
|  | H21L |
|  | H86A |
|  | H86L |
|  | H113A |
|  | H113L |
|  | H150A |
|  | H150L |
|  | H173A |
|  | H173L |
|  | H261A |
|  | H261L |
|  | H303A |
|  | H303L |
|  | H328A |
|  | H328L* |
|  | H397A |
|  | H397L |
|  | H419A |
|  | H419L |
|  | H424A |
|  | H424L |
|  | H86A-H397A |
|  | H150A-H261A-H328A |
|  | H20A-H21A-H173A |
| Rosetta Redesign 2: AAs 376-389 | L380P |
|  | Q383P |
|  | H384P |
|  | S379K |
|  | S379R |
|  | L381G |
|  | Q383D |
|  | H384K |
|  | L388G |
| Rosetta Redesign 3: Pre-fusion trimer interface | E275L R278F K279T E282L (RR Interface 1) |
|  | R278F K279T E282L (RR Interface 2) |
|  | E274I E275V R278F K279T E282L (RR Interface 3) |
|  | E274I* |
|  | E274K |
|  | E274L |
|  | E274Q |
|  | E274V |
|  | E275I |
|  | E275K |
|  | E275L |
|  | E275Q |
|  | E275V |
| Resistant to Acid-Induced Neutralisation (RAIN) | M44I |
|  | M44V |
|  | V392G |
|  | M396T |
|  | E282K* |

**Table S 1: RVG mutants designed for pre-fusion stabilisation.** Mutants were designed using a computation model of the pre-fusion RVG structure and are categorised by mutation strategy type. RAIN mutations are as previously described [4]. Where mutants are referred to by pseudonyms in figures, these are included in parentheses. *Mutants that were designed but not cloned or tested in vitro.

## Table S2

| Combinatorial Mutant | | Mutation Strategy 1 | Mutation Strategy 2 | Mutation Strategy 3 |
| --- | --- | --- | --- | --- |
| H270P_D266P | HbH Proline | | RR1 HbH |  |
| H270P_H261L |  |  | His Switch |  |
| H270P_R264I |  |  | RR1 HbH |  |
| H270P_S379R |  |  | RR2: 376-389 |  |
| H270P_M396T |  |  | RAIN |  |
| H270P_H419L |  |  | His Switch |  |
| H270P_L271Q |  |  | RR1 HbH |  |
| H270P_I268N |  |  | RR1 HbH |  |
| H270P_V272P |  |  | HbH Proline |  |
| H270P_V273P |  |  | HbH Proline |  |
| V272P_D266P |  |  | RR1 HbH |  |
| V272P_H261L |  |  | His Switch |  |
| V272P_R264I |  |  | RR1 HbH |  |
| V272P_S379R |  |  | RR2: 376-389 |  |
| V272P_H419L |  |  | His Switch |  |
| V272P_L271Q |  |  | RR1 HbH |  |
| V272P_I268N |  |  | RR1 HbH |  |
| V272P_V273P |  |  | HbH Proline |  |
| D266P_H261L | RR1 HbH | | His Switch |  |
| D266P_R264I |  |  | RR1 HbH |  |
| D266P_S379R |  |  | RR2: 376-389 |  |
| D266P_M396T |  |  | RAIN |  |
| D266P_H419L |  |  | His Switch |  |
| D266P_L271Q |  |  | RR1 HbH |  |
| D266P_I268N |  |  | RR1 HbH |  |
| D266P_V273P |  |  | HbH Proline |  |
| H261L_R264I | His Switch | | RR1 HbH |  |
| H261L_S379R |  |  | RR2: 376-389 |  |
| H261L_M396T |  |  | RAIN |  |
| H261L_H419L |  |  | His Switch |  |
| H261L_L271Q |  |  | RR1 HbH |  |
| H261L_I268N |  |  | RR1 HbH |  |
| H261L_V273P |  |  | HbH Proline |  |
| R264I_S379R | RR1 HbH | | RR2: 376-389 |  |
| R264I_M396T |  |  | RAIN |  |
| R264I_H419L |  |  | His Switch |  |
| R264I_L271Q |  |  | RR1 HbH |  |
| R264I_I268N |  |  | RR1 HbH |  |
| R264I_V273P |  |  | HbH Proline |  |
| V273P_H419L | HbH Proline | | His Switch |  |
| V273P_L271Q |  |  | RR1 HbH |  |
| V273P_I268N |  |  | RR1 HbH |  |
| H419L_L271Q | His Switch | | RR1 HbH |  |
| H270P_V272P_H419L | HbH Proline | | HbH Proline | His Switch |
| H270P_D266P_H419L |  |  | RR1 HbH | His Switch |
| H270P_H261L_H419L |  |  | His Switch | His Switch |

**Table S 2: Combinatorial RVG mutants.** The mutation strategies from which parent single mutants are derived are shown. HbH Proline = Helix-bend-helix proline mutations, RR1 HbH = Rosetta Redesign 1: Helix-bend-helix, His Switch = Histidine switch, RR2: Rosetta Redesign of Aas 376 – 389, RAIN = Resistant to Acid Induced Neutralisation.

# Supplementary Bibliography

[1] T. Müller *et al.*, “Development of a mouse monoclonal antibody cocktail for post-exposure rabies prophylaxis in humans,” *PLoS Negl Trop Dis*, vol. 3, no. 11, Nov. 2009, doi: 10.1371/JOURNAL.PNTD.0000542.

[2] H. Cao *et al.*, “A rabies mRNA vaccine with H270P mutation in its glycoprotein induces strong cellular and humoral immunity,” *Vaccine*, vol. 42, no. 5, pp. 1116–1121, Feb. 2024, doi: 10.1016/J.VACCINE.2024.01.057.

[3] T. D. Goddard *et al.*, “UCSF ChimeraX: Meeting modern challenges in visualization and analysis,” *Protein Sci*, vol. 27, no. 1, pp. 14–25, Jan. 2018, doi: 10.1002/PRO.3235.

[4] Y. Gaudin, H. Raux, A. Flamand, and R. W. Ruigrok, “Identification of amino acids controlling the low-pH-induced conformational change of rabies virus glycoprotein.,” *J Virol*, vol. 70, no. 11, p. 7371, Nov. 1996, Accessed: Aug. 17, 2021. [Online]. Available: /pmc/articles/PMC190804/?report=abstract
